# An attempt at modeling COPD epidemiological trends in France

**DOI:** 10.1186/s12931-018-0827-7

**Published:** 2018-06-27

**Authors:** Pierre-Régis Burgel, Caroline Laurendeau, Chantal Raherison, Claire Fuhrman, Nicolas Roche

**Affiliations:** 1 Respiratory Medicine, Hôpital Cochin, AP-HP, EA2511, Université Paris Descartes, Sorbonne Paris Cité, 75014 Paris, France; 2 CEMKA-EVAL, 92340 Bourg-La-Reine, France; 3 U1219 institut de santé publique, d’épidémiologie et de développement (ISPED), Service des maladies respiratoires, Université Bordeaux-Segalen, CHU de Bordeaux, 33600 Pessac, France; 4 Respiratory Medicine, Centre Hospitalier Intercommunal de Créteil, 94000 Créteil, France; 5 Service de Pneumologie, Hôpital Cochin, 27 Rue du Faubourg St Jacques, 75014 Paris, France

**Keywords:** COPD, Prevalence, Severity distribution, Projection, Epidemiological model

## Abstract

**Background:**

Anticipating the future burden of chronic obstructive pulmonary disease (COPD) is required to develop adequate public health policies.

**Methods:**

A dynamic population model was built to estimate COPD prevalence by 2025 using data collected during the most recent large general population study on COPD prevalence in France (2005) as baseline values. Sensitivity analyses were performed to test the effect of variations in key input variables.

**Results:**

The model predicted a steady increase in crude COPD prevalence among subjects aged≥45 years from 2005 (prevalence estimate: 84.51‰) to 2025 (projected prevalence: 95.76‰, + 0.56‰/yr). There was a 4-fold increase in the prevalence of GOLD grade 3–4 cases, a 23% relative increase in women and a 21% relative increase in subjects ≥75 years. In sensitivity analyses, these temporal trends were robust. Factors associated with > 5% relative variations in projected 2025 prevalence estimates were baseline prevalence and severity distribution, incidence in women and severity of incident cases, transition rates between severity grades, and mortality.

**Conclusions:**

Projections of future COPD epidemiology consistently predict an increase in the prevalence of moderate-to-very severe COPD, especially due to increases among women and subjects aged ≥75 years. Developing robust prediction models requires collecting reliable data on current COPD epidemiology.

**Electronic supplementary material:**

The online version of this article (10.1186/s12931-018-0827-7) contains supplementary material, which is available to authorized users.

## Background

To optimize the allocation of resources, healthcare authorities need consistent projections estimating the future burden of major chronic diseases [1, 2]. Chronic obstructive pulmonary disease (COPD) is recognized as one of the leading non-communicable chronic diseases in terms of prevalence, mortality, morbidity, handicap and healthcare costs [3, 4]. The burden of COPD is closely related to the prevalence of the disease and to the severity distribution among COPD patients: the vast majority of COPD-related healthcare expenses (up to 70%) are related to a small fraction (< 20%) of the patients who require hospitalizations [5, 6].

Estimating the burden of COPD has proven difficult due to marked heterogeneity among epidemiological studies [7]. Methodological differences between studies relate essentially to the variability in patient sampling, and the criteria used to define COPD and to categorize its severity. Most studies were performed in specific settings such as General Practitioner (GP) practices or healthcare centers, or in limited geographical areas. Even when their results have been adjusted to the demographic characteristics of the general population, it is never possible to ensure that estimates correspond to what would be found in the “real” general population.

COPD is mostly due to tobacco smoking, which accounts for approximately 80% of cases in industrialized countries [8]. Although the role of other environmental factors is increasingly recognized [9], smoking is anticipated to remain the main risk factor of COPD for many years in industrialized countries. Consequently, trends in smoking habits in the population are considered a major determinant of the future burden of the disease in developed countries. Another important determinant of COPD prevalence is ageing, as disease prevalence increases markedly in older subjects [10].

The purpose of the present study was to use currently available epidemiological data to develop a model predicting future trends in COPD epidemiology in France, based on currently available data and to examine how variations in the main explanatory input variables associated with COPD (e.g., ageing of population, smoking habits, incidence, current prevalence, and mortality) could affect future trends.

## Methods

### Structure of the model

#### General principles

**Fig. 1:**
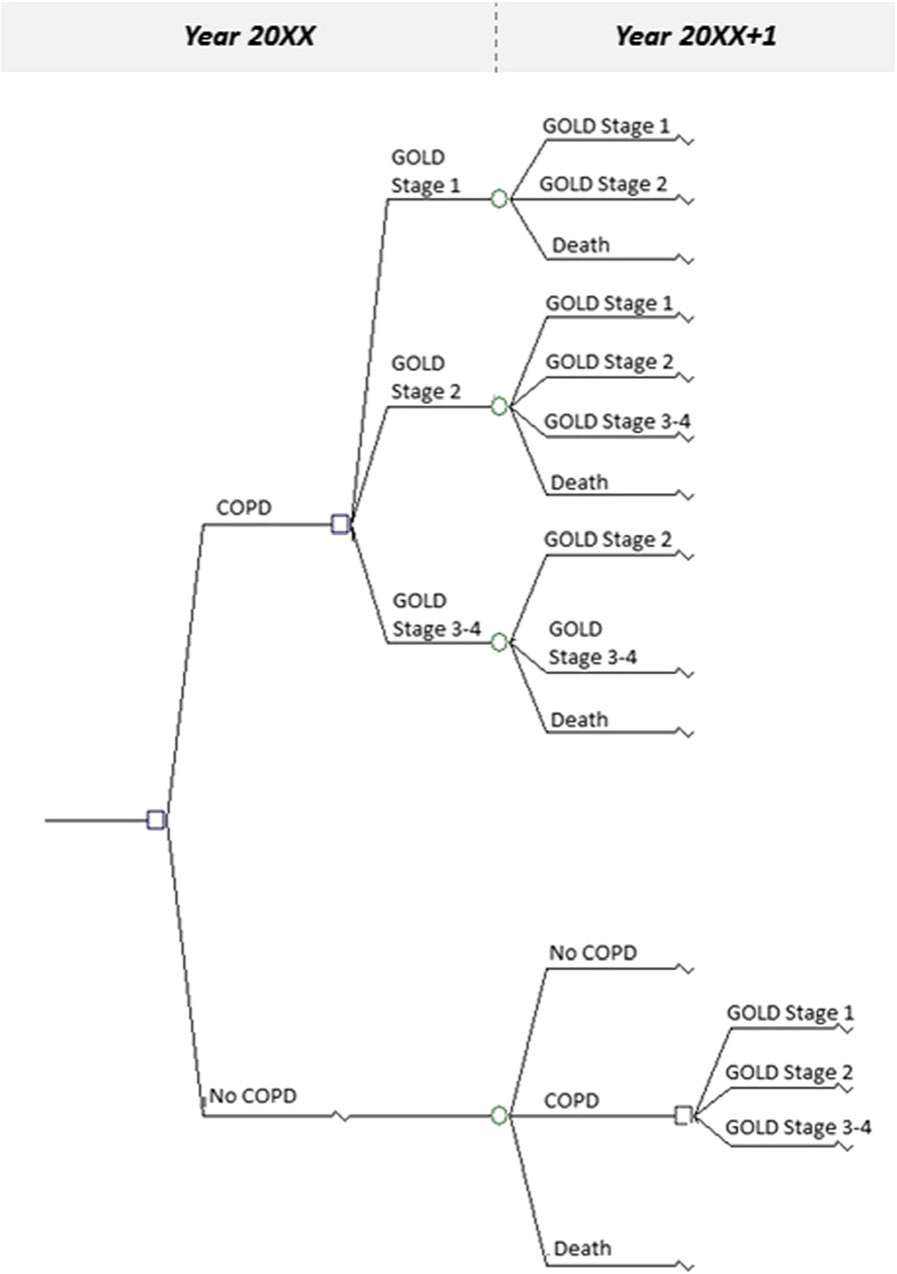
General structure of the model used to estimate the prevalence of COPD in 2025

The present model is a dynamic population model which begins in 2005, i.e. when the most recent data on COPD prevalence in the general population aged 45 years or more was collected in France [11]. This multi-state model projects the prevalence of COPD from 2005 to 2025 in the French population aged 45 years and more, using data on prevalence, incidence, mortality and progression of the GOLD-defined severity of airflow limitation. At the end of the first year (2005), the population was divided in one of three possible health states (COPD, no COPD, and Death) according to COPD prevalence and mortality rates. During all subsequent years, people moved between these states according to incidence and mortality rates. In addition to the initial 2005 population, new subjects entered the population each year: this entrant population corresponded to all persons who were aged 44 years during the previous year. The entrant population was divided in one of the above-mentioned three possible health states according to COPD prevalence rate and mortality rate at 45 years. Gender, smoking status and change in smoking status were taken into account in the model for both COPD and non-COPD populations. In the COPD population, subjects were also categorized according to GOLD-defined spirometric grades of airflow limitation and probabilities of COPD transition toward more or less severe spirometric grades [12]. Figure 1 shows the general structure of the model.

#### Definition of the COPD population

The COPD population at a given time-point is composed of three distinct components: the COPD population of the past year still alive during the considered year, the population of incident COPD in the population aged more than 44 years the year before, and the population of prevalent COPD in the population aged 44 years the past year. The COPD population was categorized according to gender, smoking status and severity of airflow limitation.

#### Data sources

To generate hypotheses used in the reference and sensitivity analyses, a systematic review of published data was performed using Pubmed database (keywords: COPD, epidemiology, incidence, prevalence, population; languages: English or French). This literature search identified original research and previous systematic reviews published after year 2000 and provided estimates of the incidence and prevalence of COPD in France and in other industrialized countries around year 2005, when the reference prevalence data was collected in France. The literature search was limited to articles in which the diagnosis of COPD was confirmed by spirometry, excluding articles that used self-reported and/or physician-reported diagnosis. Retrieved articles were reviewed by all the authors to assess the methodology and describe the source population, COPD diagnostic criteria, and population characteristics regarding age, gender, smoking status, and severity distribution of identified COPD cases. The structure, assumptions, input data and results of the analyses were the results of a consensus of a panel of expert pulmonologists, epidemiologists and statisticians (i.e., the authors of the present article).

### Input data used in the reference analysis

#### The French population in 2005

The structure of the French population as per January 1st 2005 was obtained from the French National Institute of Statistics and Economic Studies (INSEE): 25665019 (54% of women) subjects were ≥ 45 years, and 49.3% of these subjects were ≥ 65 years (Additional file 1).

#### Prevalence of COPD

The prevalence of COPD and its distribution by gender, age and smoking status was derived from the general population study performed in France in 2005 [11] among 4764 male and female subjects visiting healthcare prevention centers. After adjustment to the general population characteristics, this study estimated at 8.4% the prevalence of COPD in France. Most COPD patients were mild to moderate cases with a GOLD 1 or 2 spirometric grade of airflow limitation (51.4 and 31.5% of all COPD, respectively). Details on these data can be found in Additional file 2: 2A and 2B.

#### Incidence rates

In the absence of recent study assessing COPD incidence rate by gender and severity in France, data was derived from results obtained in other European countries during the same period as the French general population prevalence study, to ensure chronologic consistency [13–15] (see text in the Additional file 3 and Additional file 4). According to these studies, COPD annual incidence rate was 0.6% in men and 0.3% in women, 95.4% of cases were diagnosed at GOLD 1 or GOLD 2 spirometric severity grades, 64.1% were diagnosed after 65 years of age, 63.5 and 32.9% were diagnosed in current smokers and ex-smokers, respectively.

#### Transition rates

The probabilities of transition between GOLD grades were those reported by Casanova et al. in a Spanish cohort (Additional file 5) [12], while probabilities of changes in smoking status were those used in the model developed by Hoogendoorn et al. [13]. In the COPD population, the annual probability of smoking cessation was estimated at 4.7% while the probability of starting smoking again was estimated at 2.6% among ex-smokers [13]. In the non-COPD population, it was estimated that each year 3.6% of smokers stop smoking, 6.5% of ex-smokers start smoking again and 0.8% of non-smokers start smoking (Additional file 6) [13].

#### Mortality rates

Mortality rates used in the analysis were obtained by combining death rates observed in the general population (national mortality tables by age and gender) with the additional risk of death in smokers as estimated by Doll et al. [16]) and with the additional risk of death by COPD severity stage as estimated by Mannino et al. [17] (see Additional file 3), who reported a relative risk of death of 1.4 in GOLD grade 1 (versus no COPD), 2.04 in GOLD grade 2 and 2.7 in GOLD grade 3. These multiplicative risks of death relative to COPD grades were hypothesized stable with age, although they could overestimate the number of annual COPD-related deaths in elderly people.

### Sensitivity analyses

Several sensitivity analyses were performed to assess the impact of variations of the main input variables on the estimates (prevalence and trends) provided by the dynamic model. These variables were: baseline (2005) prevalence of COPD (a +/− 10% variation was applied, slightly exceeding the 95% confidence interval limits of the 2005 estimates), distribution of COPD severity (considering the possibility of a bias toward less severe cases in the studied 2005 population), age and smoking status among prevalent cases, COPD incidence rate, distribution of COPD severity among incident cases, COPD-related mortality and additional risks of COPD-related death by GOLD grade, probabilities of transition between GOLD grades and between smoking status, proportion of smokers in the French population. More details on applied variations and their rationale (e.g., data sources) are provided in the online supplement.

## Results

### Reference analysis

#### Estimation of the 2005 prevalence of COPD

According to estimates from the 2005 French epidemiological study [11] adjusted to the general population structure, the mean prevalence of COPD in people aged ≥45 years was 10.6% for males, 6.7% for females and 8.4% for the entire population (Additional file 2: 2A and 2B). Among subjects with COPD, 58.4% were in GOLD grade 1, 37.4% in GOLD grade 2, 4.2% in GOLD grade 3–4. Males represented 55.3% of the COPD population, among which 25% were current smokers and 33% ex-smokers, and age distribution was as follows: 45–54 years: 21.6%; 55–64 years: 23.8%; 65–74 years: 25.1% and 75 years and more: 29.5%. Altogether, these figures corresponded to 2,253,924 COPD cases including 94,337 with severe or very severe airflow limitation.

#### Projected evolution of COPD prevalence: 2005–2015

**Fig. 2:**
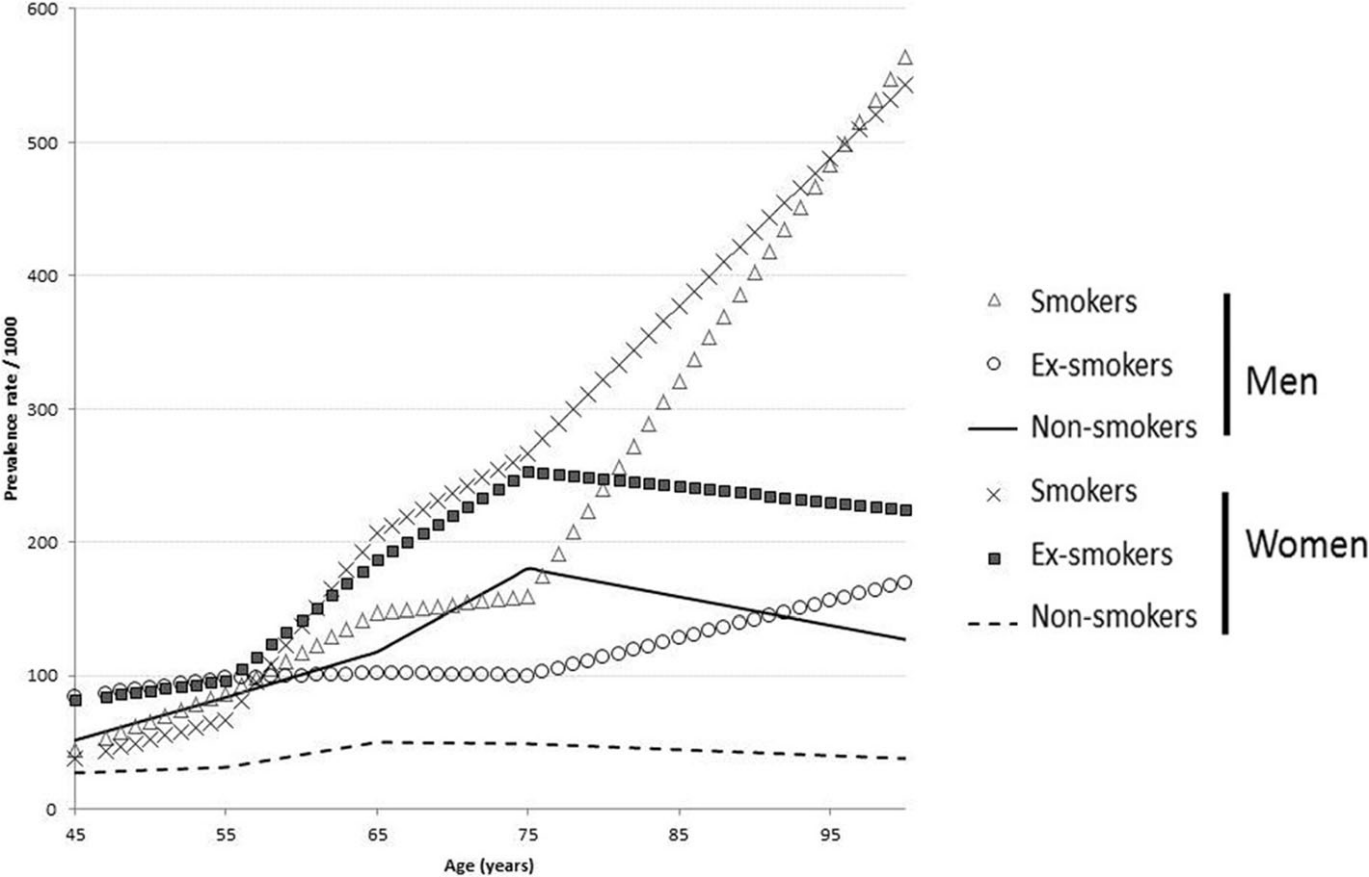
Prevalence of COPD in 2007 by gender, smoking status and age: data used as baseline values for the dynamic model

**Fig. 3:**
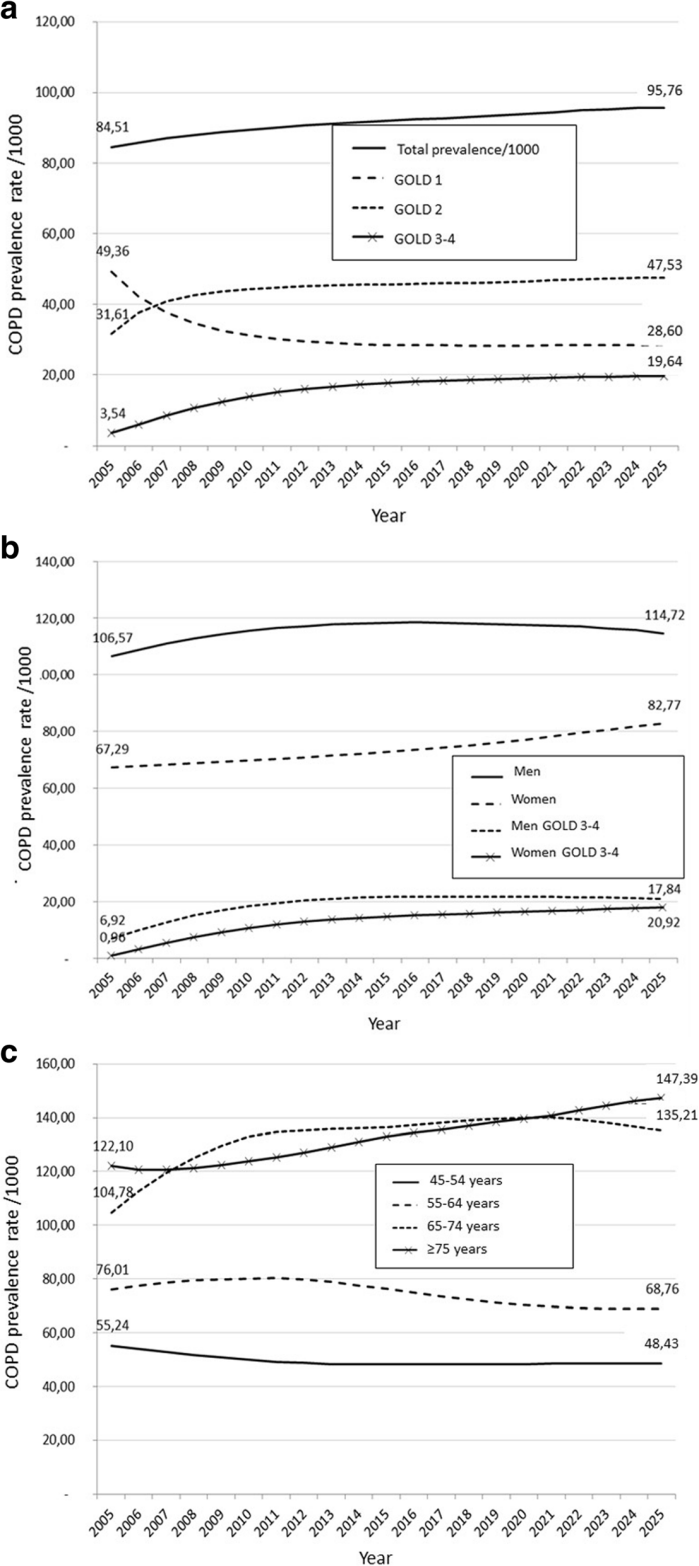
Projected trends in COPD prevalence, overall and by GOLD grade (**a**), gender (**b**) and age (**c**): results of the reference analysis

The model projected an increase in the number of COPD cases from 2,253,924 in 2005 to 2,801,650 in 2025, corresponding to an increase in prevalence of COPD from 8.4 to 9.6% during the same period (see Table 1). Figure 2 depicts projected trends in the prevalence of COPD and its distribution by GOLD grades, gender, and age according to the reference analysis are presented in Fig. 3a, b and c, respectively. Increases were shown for GOLD 2–4, women and age ranges 45–54 years and ≥ 75 years.

**Table 1:** Projected total population aged ≥ 45 years and number of subjects with COPD in France, overall and by spirometric GOLD grade, from 2005 to 2015, according to the reference analysis

Year	Number of cases	Prevalence rates/1000			
		All	GOLD Grade 1	GOLD Grade 2	GOLD Grade 3–4
2005	2,253,924	84.51	49.36	31.61	3.54
2010	2,499,336	89.53	31.17	44.42	13.94
2015	2,645,236	92.07	28.57	45.71	17.79
2020	2,746,957	93.96	28.34	46.60	19.03
2025	2,801,650	95.76	28.60	47.53	19.64

### Sensitivity analyses

Table 2 shows the main results of the sensitivity analyses regarding the respective prevalence of COPD and GOLD grades 3–4 of airflow limitation, when using alternative hypotheses. Factors associated with the largest differences between sensitivity analyses and the reference analysis were variations in baseline prevalence and severity distribution, incidence in women and severity of incident cases, transition rates between severity grades, and mortality. Other alternative hypotheses induced relative variations of less than 5%. Reducing transition rates to more severe grades of airflow limitation was associated with a marked decrease in the projected future prevalence of cases with severe or very severe airflow limitation.

**Table 2:** Summary of sensitivity analyses: prevalence rates (/1000) for COPD and severe + very severe COPD (GOLD grade 3–4) as predicted by the dynamic model for year 2025

Variable	Alternative hypothesis entered as baseline values for the sensitivity analysis	COPD		Severe+very severe airflow limitation	
		Prevalence /1000	Relative % change	Prevalence /1000	Relative % change
Prevalence	10% relative increase in 2005 prevalence (~upper limit of the confidence interval)	100.09	+ 4.5%	20.62	+ 5.0%
	10% relative decrease in 2005 prevalence (~lower limit of the confidence interval)	91.42	−4.5%	18.65	−5.0%
	Variation in the distribution of prevalent cases by severity of airflow limitation [13]	90.82	−5.2%	18.98	−3.4%
	Variation in the distribution of prevalent cases by smoking status	94.89	−0.91%	19.40	−1.2%
Incidence	10% increase in incidence	100.36	+ 4.8%	20.50	+ 4.4%
	10% decrease in incidence	91.11	−4.9%	18.77	− 4.4%
	Increase of incidence in women, reaching incidence in men	120.81	+ 26.2%	24.22	+ 23.3%
	Increase from 4.5 to 14% of the proportion of incident cases with severe or very severe airflow limitation at diagnosis	97.01	+ 1.3%	22.04	+ 12.2%
Probabilities of transition between GOLD grades	Reduction of transition rate to a more severe category, from 10 to 20 to 5%	98.41	+ 2.8%	8.92	−54.6%
Mortality [17]	Lower limit of the 95% confidence intervals of hazards ratios of COPD-related mortality by severity of airflow limitation	104.77	+ 9.4%	22.10	+ 12.5%
	Upper limit of the 95% confidence intervals of hazards ratios of COPD-related mortality by severity of airflow limitation	85.31	−10.9%	16.98	−13.5%
Probabilities of transition between smoking status	No change in individual smoking status	93.18	−2.7%	18.95	−3.5%

Percentages indicate the % of difference between each sensitivity analysis and the reference analysis. Details on alternative hypotheses can be found in the Additional files 1, 2, 3, 4, 5 and 6

## Discussion

Models developed in this study consistently predicted a modest but steady increase in COPD prevalence in France, prominently affecting women and subjects aged 75 years or more. The reference model predicted an average 0.6‰/year absolute increase in prevalence from 2005 to 2025. The variables that influenced most future trends were incidence and mortality in COPD patients. Transition rates between GOLD grades of airflow limitation had a marked influence on the prevalence of cases with severe and very severe airflow limitation.

The purpose of the present model is to allow prediction of COPD epidemiological trends over extended periods of time. Only few studies performed projections of COPD epidemiology over time. One of the first dynamic model was developed by Feenstra et al. [18] almost two decades ago and further elaborated on a few years later by Hoogendoorn et al. [13]. This model anticipated an increase in COPD prevalence for all severity stages, especially in women. In a systematic review published in 2015 by McLean et al., 6 high-quality models were identified including these two [19]. The models were designed to estimate future disease burden using trends in demographics and risk factors, Markov-type modelling and microsimulation modelling. In addition to their differences in mathematical aspects and input data, these models differ in terms of modeled output variable(s): prevalence, mortality, disability, and/or costs. These variations make it difficult to compare results between studies [7]. Therefore, sensitivity analyses represent a critical component of such modeling approaches since they allow to test the respective weight of the various input data as determinants of future disease burden. Another important aspect is external validation using actual data from longitudinal or repeated surveys. In a recent manuscript, Molinari et al. used data from hospital coding databases in France and reported increased rates of hospitalization and related-mortality [20]. Altogether, all models and surveys converge with our present study in demonstrating a high likelihood of further increase in COPD burden at a population level [3].

The initial project was planned to rely only on data collected in France, since projections were to be made for this country only, at least as a first step of the model development. In addition, it was thought that epidemiological data would be more homogeneous when restricted to a single country. However, it appeared that only a limited number of epidemiological studies were available, some of which were ancient. Thus, some of the input data were obtained using articles published in the same time frame in industrialized countries, which were available in a limited number of countries, resulting in some heterogeneity. In addition, methods of case recruitments were quite different: in one study, the studied population was recruited in health care prevention centers and results were adjusted to match the characteristics of the general population [11]. In that study, an 8.4% prevalence of COPD was found, which is consistent with the results of other epidemiological studies in developed countries. However, the proportion of patients with severe airflow obstruction was rather low, in contrast to what had been observed elsewhere [7]. The authors hypothesized that this finding could be biased by the source of the sample, since patients visiting prevention centers for a general health check-up are supposed not to be managed for some severe illness. In a European study (the European Community Respiratory Health Survey), part of the studied population was recruited in France [21]. However, only two towns participated, which makes it difficult to extrapolate to the whole French population, even after appropriate adjustments; in addition, the age structure of the population recruited in that study was very different (age range, 20–44 years). In the Confronting COPD study [22] and its follow-up study [23], to which France participated [24, 25], the diagnosis of COPD did not rely on spirometry but only on subjects-reported medical history and symptoms. Considering the possible bias in the only spirometric study available in the French general population [11] and the noticeable heterogeneity in available results regarding COPD severity distribution, it was decided to perform sensitivity analyses using data from various studies performed in developed countries at roughly the same time the French reference data were collected. These analyses suggested that, provided the initial prevalence estimates are robust, incidence and mortality rates are the main determinants of prevalence projections. Therefore, it appears important to include these variables in standard population surveillance programs, to which they do not belong at present in many countries. Our results also emphasize that regularly gathering reliable prevalence estimates at the national level is crucial to refine projections of future disease burden. Another question of interest is the contribution of occupational, domestic and atmospheric pollutants to the occurrence, severity and natural history of COPD. At present there are very few longitudinal data on how these exposures evolve by region over time and how they modulate trends in COPD epidemiology.

A surprising finding was the predicted decrease in the number patients in GOLD 1, which was counter intuitive. Because this decrease was accompanied by a comparable increase in the number of patients in GOLD 2, we speculate that this finding may be related to imbalance between the number of new GOLD 1 patients vs. a higher number of patients who transit from GOLD grade 1 to grade 2. However, we cannot exclude the presence of unidentified bias in the model.

Models such as the one developed here can produce data to inform estimations of future disease-related costs and thereby to guide future resource allocation. As previously shown, the structure of a model developed using mostly data from one country could be applied to another region provided that adequate input data is available [26]. Another application of these modeling approaches is the assessment of the cost and cost-effectiveness of long-term care [27, 28]. However, as recently emphasized in a systematic review, a major difficulty with health economics simulation models in COPD is to fully account for the disease’s heterogeneity and to include the weight of comorbid conditions [2]. Most general population epidemiological studies to not provide sufficient levels of details on the clinical characteristics of identified patients. These details can be found in observational studies or clinical trials, but corresponding populations are unlikely to represent the COPD population at large.

## Conclusion

In conclusion, epidemiological models based on current estimates can project future COPD burden. Their reliability is heavily dependent on the quality of input data. Therefore, regular surveillance of COPD epidemiology is required to obtain robust updated projections that can be used to guide healthcare policies.

## Additional files

Additional file 1: Estimated structure of the French population in 2005. (DOCX 22 kb)

Additional file 2: 2A: COPD prevalence by age, gender, and GOLD stage in the 2005 French general population. 2B: Prevalence of COPD by smoking status, gender and age in the 2005 French population. (DOCX 26 kb)

Additional file 3: Incidence rates from the literature, used to build the model. (DOCX 29 kb)

Additional file 4: Incidence of COPD in the literature published around 2005. (DOCX 22 kb)

Additional file 5: Probability of transition between GOLD stages (Casanova 2014). (DOCX 21 kb)

Additional file 6: Probability of transitions between smoking status in non-COPD subjects (1A) and patients with COPD (1B) [1]. (DOCX 61 kb)

## Abbreviations

COPD: chronic obstructive pulmonary disease
GOLD: global initiative for obstructive lung disease
GP: general practitioner
INSEE: French National Institute of Statistics and Economic Studies
