# Supplementary material for: An attempt at modeling COPD epidemiological trends in France
Source: Respir Res. 2018 Jun 27;19:130. doi: 10.1186/s12931-018-0827-7 (PMC6022451; doi:10.1186/s12931-018-0827-7)
Supplement: Supplementary file 1 — Estimated structure of the French population in 2005. (DOCX 22 kb) [file 12931_2018_827_MOESM1_ESM.docx]

**Additional file 1:** Estimated structure of the French population in 2005

| Gender | Smoking status | 45-54 years | 55-64 years | 65-74 years | 75 years and above |
| --- | --- | --- | --- | --- | --- |
| Men | Non smokers | 46% | 45% | 45% | 58% |
|  | Ex-smokers | 26% | 35% | 34% | 34% |
|  | Smokers | 28% | 20% | 21% | 8% |
| Women | Non smokers | 63% | 72% | 80% | 85% |
|  | Ex-smokers | 16% | 18% | 11% | 12% |
|  | Smokers | 21% | 10% | 9% | 3% |
