# Supplementary material for: An attempt at modeling COPD epidemiological trends in France
Source: Respir Res. 2018 Jun 27;19:130. doi: 10.1186/s12931-018-0827-7 (PMC6022451; doi:10.1186/s12931-018-0827-7)
Supplement: Supplementary file 2 — 2A: COPD prevalence by age, gender, and GOLD stage in the 2005 French general population. 2B: Prevalence of COPD by smoking status, gender and age in the 2005 French population. (DOCX 26 kb) [file 12931_2018_827_MOESM2_ESM.docx]

**Additional file 2:**

**2A**: COPD prevalence by age, gender, and GOLD stage in the 2005 French general population.

|  | Men | Women | **All** |
| --- | --- | --- | --- |
| All GOLD stages |  |  |  |
| 45-54 years | 71.7 | 44.2 | **57.9** |
| 55-64 years | 107.4 | 46.5 | **76.7** |
| 65-74 years | 132.6 | 95.7 | **112.6** |
| ≥ 75 years | 211 | 113.6 | **155.2** |
| *Total* | *109.7* | *66.3* | ***87.1*** |
| GOLD 1 |  |  |  |
| 45-54 years | 39.7 | 30.9 | **35.3** |
| 55-64 years | 49 | 38.8 | **43.8** |
| 65-74 years | 66.3 | 59.4 | **62.6** |
| ≥ 75 years | 126.6 | 78.9 | **99.3** |
| *Total* | *57.2* | *46.1* | ***51.4*** |
| GOLD 2 |  |  |  |
| 45-54 years | 29.8 | 13.2 | **21.5** |
| 55-64 years | 49 | 7.8 | **28.2** |
| 65-74 years | 54.6 | 34.7 | **43.8** |
| ≥ 75 years | 67.5 | 31.5 | **46.9** |
| *Total* | *44.6* | *19.4* | ***31.5*** |
| GOLD 3-4 |  |  |  |
| 45-54 years | 2.2 | 0 | **1.1** |
| 55-64 years | 7.9 | 0 | **3.9** |
| 65-74 years | 9.7 | 1.7 | **5.4** |
| ≥ 75 years | 16.9 | 3.2 | **9** |
| *Total* | *7* | *0.8* | ***3.8*** |

**2B**: Prevalence of COPD by smoking status, gender and age in the 2005 French population.

| Smoking status | Gender | 45-54 years | 55-64 years | 65-74 years | 75 years & more |
| --- | --- | --- | --- | --- | --- |
| Non smokers | Men | 22.2 | 30.1 | 106.3 | 64.8 |
|  | Women | 3.9 | 4.5 | 12.8 | 6.4 |
| Ex smokers | Men | 86.6 | 85.4 | 310.2 | 243.8 |
|  | Women | 33.8 | 40.1 | 204.7 | 99.8 |
| Smokers | Men | 90.4 | 168.0 | 564.7 | 564.7 |
|  | Women | 29.0 | 81.2 | 149.6 | 448.8 |
