# Supplementary material for: An attempt at modeling COPD epidemiological trends in France
Source: Respir Res. 2018 Jun 27;19:130. doi: 10.1186/s12931-018-0827-7 (PMC6022451; doi:10.1186/s12931-018-0827-7)
Supplement: Supplementary file 3 — Incidence rates from the literature, used to build the model. (DOCX 29 kb) [file 12931_2018_827_MOESM3_ESM.docx]

**Additional file 3**

**Incidence rates from the literature, used to build the model**

Hoogendoorn et al. [1], found a mean annual incidence rate in people aged>45 yrs of 6/1,000 for males and 3/1,000 for females (supplementary table E3). The distribution of severity among incident cases was estimated to be 40% for mild, 55% for moderate, 4% for severe, and 0.1% for very severe COPD. The study by Pelkonen et al. [2] provided estimates of the age distribution of incident COPD cases: 6.5% were 45-54 years old, 29.6% were 55-64 years, 56.8% were 65-74 years old and 7.3% were 75 years old and over. Smoking status distribution among incident COPD cases, as estimated by Lokke et al. [3], was 3.6% of non-smokers, 32.9% of ex-smokers and 63.5% of current smokers. Taking all these hypotheses into account, mean initial incidence of COPD in 2005 was estimated to be 4.4 per 1,000. This represents 112694 new cases of COPD in France, with 5184 with stage 3 or 4.

**Sensitivity analyses: data sources and hypotheses**

The following variations of baseline parameters were entered in the model to test its robustness and assess how these variations influence estimates of COPD prevalence and its evolution between 2005 and 2025:

- Prevalence of COPD in 2005. Sensitivity analyses were performed with the lower and upper limits of the 95% confidence intervals of the prevalence estimate found in the study by Roche et al.
- Distribution of COPD severity among prevalent cases. The reference analysis used data from Roche et al. The sensitivity analyses used severity distribution found by Hoogendoorn et al. in the Netherlands [1].
- COPD incidence rate. The reference data source was the study by Hoogendoorn et al. [1] while the alternative data source used for sensitivity analysis was the European Community Respiratory Health Survey [4].
- Distribution of COPD severity among incident cases. The main analysis was based on data from Hoogendoorn et al. [1]. Sensitivity analysis used data by Lokke et al. [3].
- COPD-related mortality. The main model used mortality rates in the general population. For the sensitivity analysis, additional risks of death observed in the population of patients with COPD were computed using French data reported by Fuhrman et al. [5].
- Probabilities of transitions between GOLD stages. An arbitrary variation of [-30%, +30%] was applied to each probability for sensitivity analyses.
- Proportion of smokers in the French population. Main data source was INSEE statistics. A sensitivity analysis was performed using data published by the INPES (National Institute for Prevention and Health Education) in 2010. This did not change projections (not shown).
- Probabilities of transitions between smoking status. The reference analysis used data from Hoogendoorn et al. [1]. The basis of the sensitivity analysis was that, for each individual, smoking status remained constant over the period of time of interest (2005-2025).

**References**

1. Hoogendoorn M, Rutten-van Molken MP, Hoogenveen RT, van Genugten ML, Buist AS, Wouters EF, Feenstra TL. A dynamic population model of disease progression in COPD. *Eur Respir J* 2005; 26: 223–233.

2. Pelkonen M, Notkola IL, Nissinen A, Tukiainen H, Koskela H. Thirty-year cumulative incidence of chronic bronchitis and COPD in relation to 30-year pulmonary function and 40-year mortality: a follow-up in middle-aged rural men. *Chest* 2006; 130: 1129–1137.

3. Lokke A, Lange P, Scharling H, Fabricius P, Vestbo J. Developing COPD: a 25 year follow up study of the general population. *Thorax* 2006; 61: 935–939.

4. de Marco R, Accordini S, Cerveri I, Corsico A, Anto JM, Kunzli N, Janson C, Sunyer J, Jarvis D, Chinn S, Vermeire P, Svanes C, Ackermann-Liebrich U, Gislason T, Heinrich J, Leynaert B, Neukirch F, Schouten JP, Wjst M, Burney P. Incidence of chronic obstructive pulmonary disease in a cohort of young adults according to the presence of chronic cough and phlegm. *Am J Respir Crit Care Med* 2007; 175: 32–39.

5. Fuhrman C, Delmas M-C, pour le groupe épidémiologie et recherche clinique de la SPLF. [Epidemiology of chronic obstructive pulmonary disease in France]. *Rev. Mal. Respir.* 2010; 27: 160–168.
