# Supplementary material for: An attempt at modeling COPD epidemiological trends in France
Source: Respir Res. 2018 Jun 27;19:130. doi: 10.1186/s12931-018-0827-7 (PMC6022451; doi:10.1186/s12931-018-0827-7)
Supplement: Supplementary file 4 — Incidence of COPD in the literature published around 2005. (DOCX 22 kb) [file 12931_2018_827_MOESM4_ESM.docx]

**Additional file 4:** Incidence of COPD in the literature published around 2005.

|  | Estimates | Source |
| --- | --- | --- |
| Gender  Men  Women | 6 ‰  3 ‰ | Hoogendoorn. Eur Respir J. 2005 |
| GOLD stage of incident cases  Stade I  Stade II  Stade III  Stade IV | 40.9 %  54.5 %  4.5 %  0.1 % | Hoogendoorn. Eur Respir J. 2005 |
| Age of incident cases  45-54 years  55-64 years  65-74 years  75 years et plus | 6.5 %  29.6 %  56.8 %  7.3 % | Pelkonen. Chest. 2006 |
| Smoking status of incident cases  Non smokers  Ex-smokers  Smokers | 3.6 %  32.9 %  63.5 % | Løkke. Thorax. 2006 |
