# Supplementary material for: An attempt at modeling COPD epidemiological trends in France
Source: Respir Res. 2018 Jun 27;19:130. doi: 10.1186/s12931-018-0827-7 (PMC6022451; doi:10.1186/s12931-018-0827-7)
Supplement: Supplementary file 5 — Probability of transition between GOLD stages (Casanova 2014). (DOCX 21 kb) [file 12931_2018_827_MOESM5_ESM.docx]

**Additional file 5**: Probability of transition between GOLD stages (Casanova 2014).

| COPD year N+1 | Stage 1 | Stage 2 | Stage 3 |
| --- | --- | --- | --- |
| COPD year N |  |  |  |
| Stage 1 | 77.2% | 22.8% | 0% |
| Stage 2 | 10.0% | 79.7% | 10.3% |
| Stage 3-4 | 1.2% | 17.0% | 81.9% |
