# Supplementary material for: An attempt at modeling COPD epidemiological trends in France
Source: Respir Res. 2018 Jun 27;19:130. doi: 10.1186/s12931-018-0827-7 (PMC6022451; doi:10.1186/s12931-018-0827-7)
Supplement: Supplementary file 6 — Probability of transitions between smoking status in non-COPD subjects (1A) and patients with COPD (1B) [1]. (DOCX 61 kb) [file 12931_2018_827_MOESM6_ESM.docx]

**Additional file 6**: probability of transitions between smoking status in non-COPD subjects (1A) and patients with COPD (1B) [1]

Non-smokers

Non-smokers

Smokers

Ex-smokers

0 %

4.7 %

2.6

%

Smokers

Ex-smokers
